# The Fingerprint of Fortified Wines—From the Sui Generis Production Processes to the Distinctive Aroma

**DOI:** 10.3390/foods12132558

**Published:** 2023-06-30

**Authors:** Rosa Perestrelo, Yassine Jaouhari, Teresa Abreu, Mariangie M. Castillo, Fabiano Travaglia, Jorge A. M. Pereira, José S. Câmara, Matteo Bordiga

**Affiliations:** 1 CQM—Centro de Química da Madeira, Universidade da Madeira, Campus da Penteada, 9020-105 Funchal, Portugal; rmp@staff.uma.pt (R.P.); teresa.abreu@staff.uma.pt (T.A.); mariangie.castillo@staff.uma.pt (M.M.C.); jorge.pereira@staff.uma.pt (J.A.M.P.); jsc@staff.uma.pt (J.S.C.); 2 Department of Pharmaceutical Sciences, Università degli Studi del Piemonte Orientale “A. Avogadro”, Largo Donegani 2, 28100 Novara, Italy; yassine.jaouhari@uniupo.it (Y.J.); fabiano.travaglia@uniupo.it (F.T.); 3 Departamento de Química, Faculdade de Ciências Exatas e Engenharia, Universidade da Madeira, Campus da Penteada, 9020-105 Funchal, Portugal

**Keywords:** fortified wines, sui generis, aroma descriptors, winemaking process

## Abstract

The fortified wines that originated in Mediterranean countries have, in common, a high alcohol content to increase their shelf-life during long journeys to northern Europe and the American continent. Nowadays, the world’s better-known wines, including Marsala, Madeira, Port, and Sherry, due to their high alcoholic content, sweet taste, and intense aromatic profile, are designated as dessert wines and sometimes served as aperitifs. This review gives an overview of the traditional vinification process, including the microbiota and autochthonous yeast, as well as the regulatory aspects of the main Italian, Portuguese, and Spanish fortified wines. The winemaking process is essential to defining the volatile organic compounds (VOCs) that characterize the aroma of each fortified wine, giving them an organoleptic fingerprint and “terroir” characteristics. The various volatile and odorous compounds found in fortified wines during the oxidative aging are discussed in the last part of this review.

## 1. Introduction

Wine is one of the oldest recorded drinks in history. Different wines, with an emphasis on fortified wines, have played a fundamental role in the culture, history, and habits of different civilizations. In Europe, fortified wines have been produced for centuries and have become an integral part of the wine culture in several countries. In fact, Port and Madeira wines, produced in Portugal, Sherry in Spain, and Marsala, Malvasia delle Lipari liquoroso and Vernaccia di Oristano liquoroso, produced in Italy, are among the most popular fortified wines in the world. European Union regulations define fortified wines, generally, as those having an acquired alcohol content by volume of between 15 and 22%, and a total alcohol content (i.e., acquired alcohol plus potential alcohol) of at least 17.5% vol. is registered for the fortification with wine-derived spirits [1].

The origin, production process, and unique sensory properties of fortified wines are closely related to the history of the people who developed and improved them over time. Madeira wine, for instance, has been produced on Madeira Island since the 15th century and is intimately tied to the Age of Discovery and Portuguese endeavors across the globe [1,2]. Similarly, Port wine, produced in the north of Portugal (in the Douro Valley), has a long history dating back to 1756, when the Douro Demarcated Region (DDR) was declared the third protected wine region in the world. It became very popular in England and throughout the English empire following the peninsular campaigns against France [1,3]. Sherry, or *vino de Jerez,* as it is known in Spain, is another popular fortified wine produced in the southern regions of Jerez de la Frontera (Cádiz) and Montilla-Moriles (Córdoba). Its production has been improved over centuries, and contemporary Sherry became more notorious when it started to be regularly exported throughout Europe, particularly to England, in the sixteenth century [1,4]. Regarding Italian fortified wines, Marsala is the most popular, being produced exclusively in the Sicily region, while other fortified wines, such as Vernaccia di Oristano and Malvasia delle Lipari Liquoroso, are less well known, being classified as niche products [5], and they will be discussed in detail in the next chapters.

As previously mentioned, the production of fortified wines involves very specific winemaking procedures that have been improved over time. These procedures result in unique types of wines that are easily distinguishable from each other. In this context, the processes used to create different types of fortified wines are very important, and several factors continuously challenge the quality of the final product. Fortified wines are expensive and are, therefore, prone to adulteration by wine producers from other regions of the world. Additionally, climate changes are affecting the soil conditions of the regions where these wines are produced, and, finally, the chemical properties of the obtained musts. For these reasons, it is of paramount importance to understand how different production processes and climate conditions affect the chemical composition and sensory properties of fortified wines.

**Figure 1:**
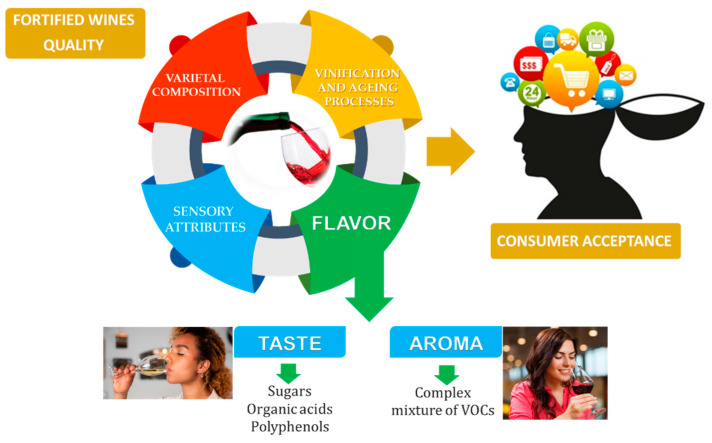
**The** important parameters related to fortified wine quality and acceptance by consumers.

Accordingly, fortified wines produced in different regions, including Portugal, Spain, and Italy, will contain very specific profiles or chemical fingerprints composed of key aldehydes and ketones, lactones, and other non-volatile compounds. Consequently, fortified wines will contain distinctive notes of nuts, spices, dried fruits, oak, fruits, and flowers, which constitute essential wine sensory attributes for consumer acceptance [1,2,3,5]. The flavour is another crucial aspect of the final product, determined by the taste attributed to sugar composition, polyphenols, and organic acids, and by the aroma related to the composition of volatile organic compounds and their chemical nature and threshold odours (Figure 1).

This review will update the current knowledge of the different aspects associated with the winemaking of the main fortified wines produced in Portugal, Spain, and Italy, namely, Madeira wine, Port wine, Sherry, and Marsala, which are responsible for the typicity and organoleptic character attributed to these wines. Emphasis will be given to the contribution of the fermentative microbiota and winemaking procedures to the distinctive chemical fingerprints of these wines. Finally, an outlook on the future of fortified wines, with potential changes in the production processes, the emergence of new types of fortified wines, and the challenges and effects of climate variations on the production and quality of fortified wines, will be discussed.

## 2. The Production of Fortified Wines—Patterns and Specificities

**Figure 2:**
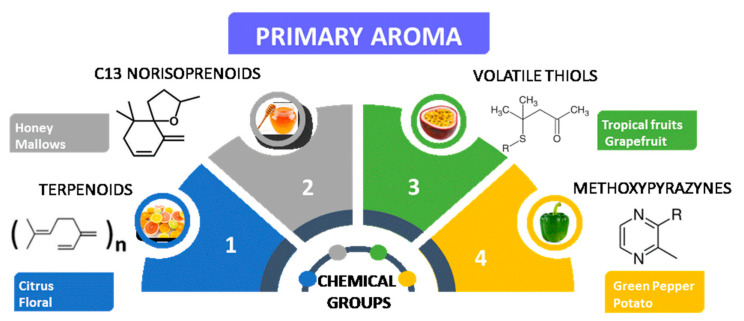
**The** most important chemical groups responsible for the wine’s primary aroma and the corresponding odour descriptors.

The production of fortified wines involves the addition of distillates, spirits, and alcohol of vinicultural origin, typically brandy, to wine during or after the fermentation process. This addition of alcohol not only increases the alcohol content of the wine, but also stops the fermentation process, leaving residual sugars in the wine, which gives it its characteristic sweetness. The specifics of production can vary depending on the type of fortified wine being made, but there are some general patterns and specificities that are common to most fortified wines [5]. One pattern in the production of fortified wines is the use of specific grape varieties. Different grape varieties have different flavor profiles and sugar levels, which can impact the final flavor of the wine. Another pattern is the use of specific production methods. Specificities in the production of fortified wines can also vary depending on the region and producer. Fortified wines are often aged for several years or even decades in oak barrels, which can impart additional flavors and aromas to the wine. The specific patterns and specificities of production can vary, depending on the type of fortified wine being made, but they all contribute to the unique and complex flavors that are characteristic of fortified wines [6]. The primary wine aroma is defined by several chemical groups, including esters, alcohols, and terpenes. Terpenoids, C13 norisoprenoids, volatile thiols, and methoxypyrazines are among the most relevant families of compounds responsible for the primary odor descriptors found in wine (Figure 2) [1].

The grape varieties, fermentation, and ageing processes are the most critical steps in defining the typicity of each fortified wine [2]. The grape varieties provide the raw material that the microbiota, including different types of yeast and bacteria, will use during the fermentation process. As a result, different secondary metabolites will be released into the wine, impacting the properties of the final product. Fortified wines are often aged and stored for long periods under different environmental conditions, adding complexity to the flavours and aromas of the wine [1,2,3,4,5].

### 2.1. The Microbiota of Fortified Wines’ Fermentation

In winemaking, the microbiota is responsible for the biotransformation of grape must into wine by alcoholic and malolactic (if necessary) fermentation process [7]. Both play an important role in the quality and typicity of wines and may occur spontaneously by the action of autochthonous microflora or by inoculation of starter cultures [7,8].

**Figure 3:**
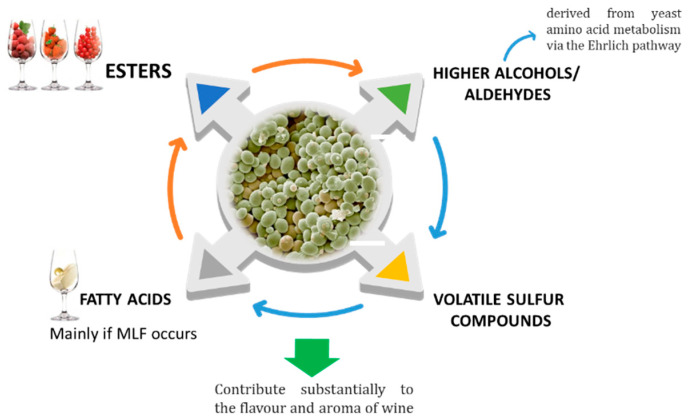
Main secondary metabolites produced by yeast metabolism.

The microbiota in the grape must is composed mostly of yeasts. During alcoholic fermentation, yeasts catabolize hexoses, not only into ethanol and carbon dioxide, but also in many volatile, semi-volatile, and non-volatile organic metabolites, such as fatty acids, esters, higher alcohols, aldehydes, and volatile sulphur compounds (Figure 3), whose diversity and proportion in the wine are related with the yeast’s species and its interactions [7,8].

By convention, the yeast wine is divided into *Saccharomyces* and non-*Sacharomyces*. The *Saccharomyces* genera, especially *Saccharomyces cerevisiae,* is the most important specie in winemaking, due to fermentative, organoleptic, and technological performance. In the last years, the positive organoleptic contribution of non-*Saccharomyces* species has been reported [9,10,11]. The nomenclature describes around 20 yeast genera with oenological interest, namely, *Aureobasidium*, *Bullera*, *Brettanomyces/Dekkera*, *Candida*, *Cryptococcus*, *Debaryomyces*, *Hanseniaspora/Kloeckera*, *Kluyveromyces*, *Metschnikowia*, *Pichia*, *Rhodotorula*, *Saccharomyces*, *Saccharomycodes*, *Schizosaccharomyces*, *Sporobolomyces*, *Sporidiobolus*, *Torulaspora*, *Williopsis,* and *Zygosaccharomyces* [7,12,13].

The autochthonous microbiota in the grapes must come naturally from the yeast population resident in the grape skin (pruine) and, indirectly, from the microbiota of the wine cellar surfaces. Generically, both environments present yeast species common to the wine production process through all regions. Nevertheless, the diversity (genera/specie/strain) and proportions are related to the geographical location and edaphoclimatic conditions of vineyards, as well the harvest and pre-fermentative operations in the wine cellar [7,12,13,14].

The major factors modulating the microbiota diversity highlight the geographical location of vineyards, such as insular, peninsular, river, mountain, and/or inland location, agronomics methods, including grape variety, vine training, irrigation, fertilization, and application of phytopharmaceuticals, and climatic conditions, such as temperature, rainfall, and humidity. Then, operations during harvesting should also be considered. This includes manual or mechanical harvest, the volume of the harvest box and transport containers, and time of transport to the wine cellar, as well as the ripeness, temperature, and the health of grapes. Finally, pre-fermentative operations in the wine cellar, such as the crushing method, sulfite and enzyme additions, temperature control, and clarification of the grape must. It should be noted that, during harvest and pre-fermentative operations, the contact of the grapes/grapes must be with different surfaces, providing the transfer of other yeasts to the grape must [8,12,15].

In a general sense, the autochthonous microbiota in the grape must varies between species that are anaerobic and aerobics, obligatory, facultatively anaerobic, weak, vigorously fermentive, and ethanol-intolerant [12,14]. The spontaneous alcoholic fermentation starts with non-*Saccharomyces* genera in major proportion, namely, *Hanseniaspora* and *Kloeckera,* which represent between 50 to 75% yeast population on grape skin, followed by the genera *Candida*. Additionally, other genera in lower percentages, such as *Cryptococcus*, *Brettanomyces*, *Metschnikowia*, *Kluveromyces*, *Pichia*, *Hansenula,* and *Rhodotorula* can be grown too. Nevertheless, this population decreases drastically and disappears due, particularly, to osmo-intolerance, followed by alcohol intolerance and low pH [7,12,13,14,16].

*Saccharomyces* genera are naturally found in a minor percentages in the grape (skin and must), and they start their exponential growth 20 h after the beginning of spontaneous alcoholic fermentation, dominating the process from the third to the fourth day, coinciding with the tumultuous fermentation phase. The *Saccharomyces* genera domine the fermentative process until the end, whereas they “colonize” the wine cellar surfaces [7,14].

#### 2.1.1. Portuguese

Madeira and Port wines are produced by spontaneous alcoholic fermentation, carried out by autochthonous yeast from vineyards and wine cellars. According to the sweetness level of the wine intended, the fermentation can be interrupted in the early stage, between a few hours until two days for the type of sweet wines [6,17], the middle stage of fermentation produces medium-sweet Madeira wines, and the last stages give way to Madeira medium-dry and dry wines [17].

The identification of species that participate in the spontaneous alcoholic fermentation of Port and Madeira wines has a few studies [17,18]. The type of sweet Port wines is carried out predominantly by *Hanseniaspora uvarum*, followed by *Lachancea thermotolerans* and *Metschnikowia pulcherrima*, which showed a high proportion (~89%) in a yeast population in laboratory wine fermentations assay. *S. cerevisiae* showed dominance in the last stage in microfermentations of certified grape must from Madeira wine cellars [18], with 8% intraspecific variability [18]. On the other hand, a microvinification assay from vineyard samples showed a dominance of *H. uvarum,* followed by *S. cerevisiae* in medium-dry Madeira wine production.

#### 2.1.2. Spanish

Sherry wines are produced by the complete alcoholic fermentation of grape must, followed by biological ageing in Fino and Amontillado types [19,20]. The grape must be inoculated by adding a variable portion of another grape must in the tumultuous phase of alcoholic fermentation, through the technique called “*pie de cuba*”, which, can be from spontaneous fermentation, and predominantly from grape must inoculated with selected dried wine yeast [19].

The dominance of *S. cerevisiae,* along with alcoholic fermentation, was reported, followed by *H. uvarum* and *Candida stellata*. Nevertheless, these were found in minor percentages and in the early stages of the process [19]. During biological ageing, the veil of flor results mainly from aggregations of yeast. *S. cerevisiae* is the most abundant. However, species such as *Torulaspora delbrueckii*, *Zygosaccharomyces rouxii*, *Z. bailii*, *Wickerhamomyces anomalus*, *Pichia membranaefaciens*, *Rhodotorula mucilaginosa*, *R. minuta*, species of genera, *Candida*, *Hansenula,* and the negative species *Dekkera bruxellensis* (anamorph *Brettanomyces bruxellensis*) were identified [19,20].

#### 2.1.3. Italian

Although the Italian disciplinaries for fortified wines are detailed, in certain cases, producers have discretion about the use of fermentation technologies, such as fermentation temperature and whether fermentation occurs using autochthonous vineyard yeast or yeast prepared in a laboratory [21]. Despite that, starter cultures selected from autochthonous yeasts prevent the consequent risk of loss of wine peculiarities and the so-called “terroir” characteristics [22]. Taking Marsala wine as an example, considered one of the most typical and consumed fortified wines in Italy, its traditional fermentation is initiated by inoculated alcohol-resistant yeasts, such as *Saccharomyces bayanus* at a controlled temperature between 18 and 20 °C [23]. Moreover, the fermentation is supported by autochthonous yeasts, such as *Saccharomyces cerevisiae* strains, present in different vineyards. A study conducted on the yeast ecology of the Sicilian Grillo grapes, used as a base for the gold and amber Marsala wines, recognized 51 different strains of *Saccharomyces cerevisiae*, in which 14 autochthonous strains revealed a technological potential for vinification. In addition, *Hanseniaspora uvarum*, *Metschnikowia pulcherrima*, *Aureobasidium pullulans*, *Pichia kudriavzevii*, and *Candida zemplinina* were isolated at high concentrations on grapes and musts after pressing [24].

### 2.2. Fortified Wines Winemaking

#### 2.2.1. Portuguese Fortified Wines

##### Madeira

Madeira wine is made on the volcanic island of Madeira, located off the coast of Portugal in the Atlantic Ocean. Madeira wine is made from a variety of grape varieties, Malvasia, Boal, Sercial, Verdelho (white varieties) known as noble varieties, and Tinta Negra (red variety). Tinta Negra is the most representative cultivar used in the winemaking of Madeira wines (around 80% of total production) [6,25]. Brandy is added to Madeira wine to stop the fermentation process and to increase alcohol content [5]. The addition of natural grape spirit occurs to obtain an ethanol content of 18% and 22% (*v*/*v*). Based on the fermentation time, wines with different sugar content will be obtained, such as dry (sugar content expressed as 49.1 to 64.8 g glucose per L, obtained from Sercial), medium dry (64.8 to 80.4 g/L, obtained from Verdelho), medium sweet (80.4 to 96.1 g/L, obtained from Boal), and sweet (96.1 to 150 g/L, obtained from Malvasia), and different glucose levels ranging from dry (till 25 g/L, fermented to low sugar levels) to sweet (130 g/L, partial fermentation) are observed in different wines. After the fermentation process, some wines undergo an ageing process in oak casks, in cellars, with a humidity level ranging from 70% to 75% (at >30 °C), while most wines go through a thermal maturation called “*estufagem*” [26]. During this process, the wine is placed in large coated vats and the temperature is increased at about 5 °C per day and maintained at 45–50 °C for three months. During “*estufagem*” complex reactions occur as the result of several underlying physical, chemical, and biochemical mechanisms promoted by dough heating, which are essential for the development of the typical aroma, taste, and colour of Madeira wines. After this treatment, the wine is allowed to undergo a maturation process in oak casks for a minimum of three years. 

In the other way some Madeira wines suffer an ageing process, from 3–20 years or longer, in cellars at 30–35 °C by sun influence, and a humidity degree higher than 70% [27]. The ageing process in oak casks is essential to the singular sensorial characteristics of Madeira wine. According to the age, the Madeira wine is classified as a vintage (a specific year of aged in casks, 17, 18, 19, and 20 years old) and blend (an average ageing period of 3, 5, 10, or 15 years old, and are called Finest, Reserve, Old Reserve, and Extra Reserve, respectively) [6].

##### Port

Port wine, also known simply as “Port”, is a fortified wine that is exclusively produced in the Douro Valley region of Portugal. It is made from a blend of indigenous grape varieties, including Touriga Nacional, Touriga Franca, Tinta Roriz, and Tinta Barroca. After the fermentation process begins, brandy is added to the wine to stop the fermentation, resulting in a wine that is both sweet and high in alcohol content. Port wine can be either red or white and is typically aged in oak barrels for extended periods of time, sometimes up to several decades [3]. The aging process can give Port wine complex flavors of berries, chocolate, caramel, and spices [26]. These red wines typically undergo long periods of aging (>four years), either through bottle aging (Vintage category) or barrel aging (Tawny category) for up to 60 years and even longer. During this maturation period, the color and wine aroma undergo some significant changes, caused by the increasing and decreasing levels of some of the chemical constituents. These changes become more pronounced with extended aging. The age of the product is related to the value, leading to a popular Portuguese expression, concerning Port wines of “the older, the better”. The changes in the aromatic profile that occur during aging are the result of several underlying reactions, which will be explained in the following chapter [28].

#### 2.2.2. Spanish Fortified Wines

Spain is renowned for its production of several types of fortified wine, including Sherry, Málaga, and Priorat [26]. Each of these wines has its own unique production process and flavour profile. Sherry, produced in the Andalusian region of Spain, is made primarily from the Palomino grape, although other varieties, such as Pedro Ximénez and Moscatel, are also used. Sherry is aged in a barrel system called the “criaderas” and “solera” system, in which younger wines are blended with older wines to create a consistent flavour profile. Sherry is also protected by a layer of yeast called “flor”, which gives the wine its distinctive nutty flavour. Sherry comes in a range of styles, from the dry and light Fino, Amontillado, and Oloroso to the sweet and dark Pedro Ximénez [4,29]. Produced in the southern region of Andalusia, Málaga is made from several grape varieties, including Pedro Ximénez and Moscatel [30]. Málaga is a sweet and fortified wine with flavours of raisins, caramel, and honey. It is usually aged in oak barrels for several years [26]. Priorat, produced in the Priorat region of Catalonia, is a red fortified wine made mainly from the Grenache and Carignan grape varieties. Priorat is aged in oak barrels for several years and has a complex flavour profile with notes of black fruits, chocolate, and spices [26]. In addition to these three types of fortified wine, Spain also produces several other fortified wines, including Montilla-Moriles, a sherry-style wine produced in the Montilla-Moriles region (Córdoba), and Rueda Dulce, a sweet and fortified wine produced in the Rueda region (Valladolid). Overall, Spanish fortified wines are highly regarded for their complexity and unique flavour profiles. They are often served as aperitifs or after-dinner drinks and can be paired with a range of foods, from savoury snacks to sweet desserts [5].

The sherry brand of wine is traditionally made from white grapes that are grown near the city of Jerez de la Frontera in Andalusia (the south of Spain) [29]. The coastal lands and the typical Mediterranean climate of this renowned wine-producing area in southern Spain, together with its aging practices, are key factors in achieving the highly desired organoleptic features of its oenological products. However, the maturation of sherry wines, until recently, was limited to the so-called “sherry triangle”, that is, the three cities: Jerez de la Frontera, Sanlúcar de Barrameda, and El Puerto de Santa Maria [29]. Sherry wines are considered among the most highly appreciated products in the world of oenology [4].

#### 2.2.3. Italian Fortified Wines

##### Marsala

Marsala represents one of the first fortified Italian wines, born out of sheer serendipitous coincidence, which has enjoyed over 200 years of worldwide success.

The Marsala wine derives from the homonymous town of Marsala in the province of Trapani. Generally, the southwest coast of Sicily is a land of sea renowned for its local grape varieties cultivated in the so-called “Fascia del Sole” (sunbelt), between the 34th and the 43rd parallels [23]. Due to the region’s dry environment and scarcity of water, grapes fail to fully mature, producing Marsala with high sugar content and little acidity [31].

The history of this fortified wine started in 1773 when John Woodhouse, an English merchant, left Liverpool to reach Sicily to buy the so-called “Barilla”, a kind of ash extracted from salt-tolerant plants, rich in carbonate and sodium sulfate, used for soap production [32]. For the first time, Woodhouse tasted “Perpetuum” (perpetual), a sweet wine matured in wooden barrels, usually kept by the locals for special occasions. During barrel ageing, a small quantity of wine is removed and replaced with a younger one. This “perpetual” operation is repeated frequently throughout the year and for several years, a method quite similar to the “soleras” used in Spain for Sherry. In that period, the Perpetuum wine was much cheaper than the Spanish and Portuguese wines. In the same year, Woodhouse organized the first consignment from Trapani to Liverpool of fifty 412-litre pipes of wine fortified with brandy, to preserve it during the sea trip. The Marsala wine was successful enough to lead Woodhouse to invest in lands and vineyards to start industrial production. Worldwide diffusion began only in the first years of the 19th century, thanks to the ability of the Italian entrepreneur Vincenzo Florio, a native of Bagnara Calabra, who founded his winery called Cantine Florio [33].

The Marsala was the first Italian wine to receive DOC recognition (“Designation of Controlled Origin”) with territorial specificity and special protection [34]. According to the production disciplinary, published in 1969, there are 29 different types of Marsala wines, distinguished by ageing time, colour, and sugar content [23]. These 29 types can be grouped into two main categories:

- Marsala Vergini: these wines are obtained from white grapes and added, after fermentation, with ethanol or wine brandy. Depending on the ageing period, the Marsala Vergini wines can be named Marsala Vergine (at least five years) or Marsala Vergine Riserva (at least ten years).
- Marsala Conciati: This category includes 27 different varieties of Marsala wines. After the fermentation, the production process provides the addition of wine brandy, cooked must, concentrated must, and so-called “mistella”, obtained by the addition of wine brandy to the must. The addition of these ingredients is essential to giving them the characteristic taste and colour. The Marsala Conciati wines must be subject to ageing to obtain the Marsa-la Fine (at least one year), Marsala Superiore (at least two years), and Marsala Superiore Riserva wines (at least four years).

A further classification is adopted according to the sugar content into *dry* (<40 g/L), *semi-dry* (40–100 g/L), and *sweet* (>100 g/L), as well as according to the colour into *oro* (gold), *ambra* (amber) and e *rubino* (ruby).

The disciplinary establishes that the Grillo, Catarratto, and Damaschino vineyards are used for the gold and amber Marsala wines, while, for the darker variety Rubino, the permitted ones are Nero d’Avola, Perricone, and Nerello Mascalese.

Except for Marsala Fine, which contains a minimum alcoholic content of 17.5%, all the other varieties are characterized by an alcohol level of at least 18%.

##### Vernaccia di Oristano Liquoroso

Vernaccia di Oristano liquoroso is a fortified wine similar in style to Sherry [35]. Local reports say that the cultivation of the vineyards of Vernaccia, also called Garnazza or Grenaccia, dates back to the Roman Empire, which defined them as “vernaculum” [36]. The Controlled Origin Designation (DOC) was obtained using white grapes farmed in the province of Oristano, in Sardinia. The Vernaccia di Oristano production is controlled by a national disciplinary rule issued after a President’s Decree in 1971, which describes four types of wine: Vernaccia di Oristano, “Vernaccia di Oristano” superiore, “Vernaccia di Oristano” riserva, and “Vernaccia di Oristano” liquoroso. The latter represents the fortified one, obtained from the base wine Vernaccia di Oristano. After being crushed and destemmed, the must is subjected to a high fermentation temperature until the resultant wine contains small amounts of residual sugars. The wine is aged for at least two years in oak or chestnut barrels before being fortified with brandy until reaching a final alcoholic content of 16.50% vol [37]. During barrel ageing, this wine undergoes biological and controlled oxidation caused by the formation of a natural yeast biofilm on the wine surface, called “flor velum” [38]. The wine has a smooth and velvety texture, often with a slightly saline note, reflecting the influence of the nearby coastal environment.

##### Malvasia delle Lipari Liquoroso

Malvasia delle Lipari liquoroso is a fortified wine prepared using Malvasia delle Lipari DOC as the base wine. The grapes “Malvasia”, employed for the production of the Malvasia delle Lipari wine, must be grown in the Aeolian Islands archipelago, located off the coast of Sicily [39]. Among the seven islands, vineyard cultivation is presently common on the islands of Salina, Lipari, and Vulcano, known for their volcanic soils characterized by a predominantly sandy fraction and high permeability. The production of Malvasia delle Lipari liquoroso involves a combination of winemaking techniques. The grapes are harvested when they are fully ripe and then left to dry in the sun or in well ventilated rooms to concentrate their sugars and flavours [40]. Once dried, the grapes are pressed, and the resulting must must be fermented. Fermentation is halted before completion by adding grape spirits, which increase the alcohol content and preserve the natural sweetness of the wine. According to the production technique defined by Regulation D.P.R. 20/09/1973, the base wine is fortified to increase the alcohol content from 12.50% vol to 20.00% vol. The Malvasia delle Lipari liquoroso is then refined for at least six months to obtain a much more aromatic amber wine. This allows for fruity aromas and scents reminiscent of apricot and peach [41]. The volcanic soils and unique microclimate of the islands contribute to the distinct character of Malvasia delle Lipari liquoroso, which is typically enjoyed as a dessert wine or digestif and is often served slightly chilled.

## 3. The Chemical Fingerprint of Fortified Wines

The chemical fingerprint of fortified wines is very complex and fascinating, being constituted by several hundred volatile and non-volatile chemical groups, such as terpenoids, pyrazines, esters, alcohols, acids, furanic compounds, phenolic compounds, and organic acids, among others. These chemical groups were present in fortified wines at different volatilities, polarities, and concentration ranges, from a few ng/L to mg/L [5]. However, the quality of wine also depends on several parameters, such as grape variety, vineyard location, terroir, and vinification conditions (e.g., fermentation, ageing), among others [5]. In the following section, the main volatile organic metabolites (VOMs) found in the most famous fortified wines (e.g., Marsala, Madeira, Porto, Sherry) will be reported, as well as the potential odorants identified in these wines.

### 3.1. Portuguese Fortified Wines

**Figure 4:**
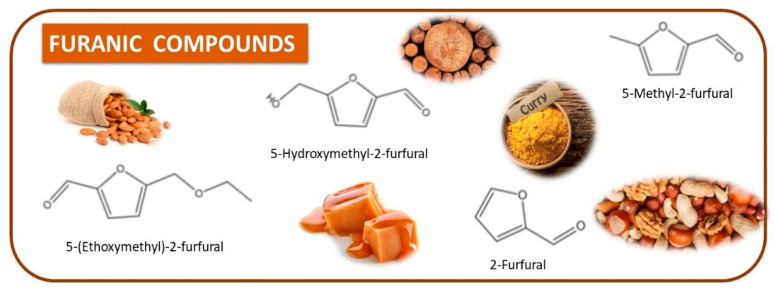
**The** chemical structures and odour descriptors of the most important furanic compounds found in Madeira wines.

Fortified Portuguese wines, such as Porto and Madeira, are known for their distinctive flavours and aromas, which are the result of a unique winemaking process that includes *estufagem* and oxidation. The aromatic complexity of Portuguese fortified wines has been extensively studied [3,28,42,43,44,45,46,47,48,49,50,51,52,53,54], and the most recent and important achievement achieved in these studies will be reported. During the ageing process, significant changes in the volatilomic profile of fortified wines occur due to the formation of new VOMs and the breakdown of existing ones. During the early stages of ageing, the wine develops fruity and floral characteristics, including VOMs belonging to esters and terpenoid chemical families. As the wine ages, these fruity and floral odours give way to more complex and intense odours, such as those linked with almond, caramel, nutty, curry, wood, and spice odours, as shown in Figure 4.

Pereira et al. [53] observed that accelerated ageing promotes the development of VOMs, such as phenylacetaldehyde, β-damascenone, and 5-(ethoxymehtyl-2-furfural), whereas other VOMs responsible for floral and fruits odours (e.g., α-terpeniol, linalool) of some Madeira wines disappears of the thermal process. Perestrelo et al. [46] observed that storage conditions promote the overall aroma of Madeira wines, as 14 VOMs appear during the storage as a result of the Maillard reaction, Strecker degradation caramelization, and microbial activity. Moreover, these VOMs contribute significantly to Madeira wine aroma complexity in relation to caramel, dried fruit, wood, spice, and toast. The aroma pattern of Madeira wines was established by Campo et al. [55], using gas chromatography–olfactometry (GC–O), and the results obtained showed that Madeira wines lack the most crucial varietal aromas (e.g., linalool, methoxypyrazines), but they are rich in wood released aroma (e.g., sotolon, phenylacetaldehyde). Silva et al. [49] studied the influence of forced ageing on Madeira wine using GC–O, and several Maillard byproducts were detected, namely, 2-furfural, 5-methyl-2-furfural, methional, sotolon, and phenylacetaldehyde. Perhaps 2-furfural and 5-methyl-2-furfural are quantitatively significant in Madeira wines, but no contribution to the overall aromas was verified due to their high odour thresholds (OTs). On the other hand, sotolon was reported as a key odorant of aged wines due to its high concentration and low OT (few µg/L) [42,55].

Other important odorants of Madeira wines aged in oak casks were butyrolactone, pantolactone, and *cis*- and *trans*-whisky lactone. In Port wines, β-damascenone, β-ciclocitral, β-ionone, branched aldehydes, and 2-alkenals isomers were found to be responsible for their aromatic complexity [48,56]. Moreover, it has also been reported that sotolon is one of the most significant odorants in Port wines. In another study, it was observed that older, when compared to younger, Port wines showed a lower content of sulphur compounds responsible for cauliflower, butter, and French bean odours [28]. The unique characteristics of Portuguese fortified wine ageing contribute to the wine’s complexity and richness, making it a sought-after and prized beverage among wine enthusiasts (Table 1).

### 3.2. Spanish Fortified Wines

Acetaldehyde has been reported as a crucial component of Sherry wines [57] and is associated with the pungent odours typical of Fino wine [58]. This VOM is a precursor of a diversity of VOMs involved in Sherry aromas, such as acetoin (buttery odours), 1,1-diethoxyethano (green fruit and liquorice odours), and sotolon (nutty, curry, and candy cotton odour). From the VOMs released from wood, special attention was given to sotolon lactone, which is responsible for the nutty odours of Sherry wines, and its concentration increased significantly during oxidative aging [59]. 1,1-Diethoxyethane, and *Z*-whisky lactone have also been proposed as potential biological ageing markers, since their concentrations increase with the ageing period [60]. Moreover, several studies have been conducted to evaluate the effect of different woods (e.g., wood type, the origin of the wood) on the volatilomic composition and organoleptic properties of these fortified wines [61,62,63]. According to Simón et al. [64], Spanish oak is suitable to age red wine, as it provides intermediate or similar organoleptic features to French and American oaks. Moreover, the concentration of hexyl acetate, ethyl pentanoate, and ethyl octanoate responsive to fruity and floral notes of aged Spanish wines decreased with ageing, except for the wines aged in French oak casks, where their concentrations, along with other VOMs, such as isoamyl acetate and isobutyl acetate, increased during ageing [62].

The aromatic complexity of Sherry wines, mainly Fino wines, has been extensively studied [57,60,65,66,67,68,69,70,71]. Acetoin is a VOM with aromatic significance in Sherry wines responsible for the bitter odours of Fino wines. The reduction in the acetoin originates 2,3-butanediol, another VOM involved in the aroma of Sherry wines [57]. Zea et al. [67] studied the influence of flor yeasts and wood on the aroma profile of Fino wines subjected to biological ageing. The data obtained showed that fruity, fatty, and spicy odours were strongly correlated to the aroma profile of Fino wines subjected to biological ageing, whereas chemical, floral, balsamic, and vegetable showed a poor correlation. The authors observed that the fruity (e.g., acetaldehyde, ethyl octanoate, ethyl acetate, sotolon, 1,1-diethoxyethane), spicy (e.g., eugenol, sotolon, Z-oak lactone, 4-ethylguaiacol), and fatty series were the ones most strongly contributing to the aroma profile of Fino wines under biological ageing, while the chemical, balsamic, vegetable, empyreumatic, and floral series, in combination, contributed in low proportions. On the other hand, fruit odours were poor in Amontillado wines due to a lower concentration of 1,1-diethoxyethane and ethyl butanoate. Fino and Oloroso wines can be distinguished from Amontillado wines through 1,1-diethoxyethane, isobutanol, phenethyl alcohol, ethyl butanoate, ethyl benzoate, isobutyl isobutanoate, isoamyl laurate, and E-nerolidol [65]. This implies that their origin may be connected to the oxidative ageing process that characterizes Oloroso wine, and for this reason, they are not present in the volatilomic profile of Amontillado wines, which undergo a subsequent oxidative ageing procedure after the first biological ageing step. β-citronellol and β-ionone are other VOMs with a significant impact on the aromatic profile of aged Sherry wines, and their presence is responsible for citrus and balsamic notes, even at low concentrations (few µg/L) [66,67,68].

### 3.3. Italian Fortified Wines

Italian fortified wines are a varied group of wines that comprise various styles, each with its own distinctive flavour and aroma profile. The volatilomic profile composition of Italian fortified wines can be affected by several factors, such as the grape variety, the winemaking, and the ageing process used. Some of the most well known Italian fortified wines include Marsala and Vernaccia di Oristano liquoroso. However, the literature data related to the volatilomic profile and odorant impacts of Italian fortified wines is very limited. Dugo et al. [72] used two-dimensional gas chromatography, coupled with time-of-flight mass spectrometry (GC × GC–TOFMS), to elucidate the volatilomic profile of four Marsala wines with different ageing characteristics (“fine”, “superiore secco”, “superiore riserva”, and “vergine”). A total of 128 VOMs were identified, belonging mainly to esters, alcohols, ketones, and aldehydes. The volatilomic profile of Marsala includes VOMs, such as acetaldehyde, ethyl acetate, ethyl hexanoate, and furfural, which give rise to fruity and nutty odours. Moreover, an attenuated total reflectance Fourier transform infrared (FTIR-ATR) method, in tandem with multivariate analysis of specific spectral areas of the sample, was developed by Condurso et al. [34] to characterize the different categories of Marsala wines based on production technology, ageing, and sugar concentration.

On the other hand, Petretto, Urgeghe, Cabizza, and Del Caro [35] investigated the volatile profile of the Sherry-like white wine Vernaccia di Oristano from Sardinia. The data obtained determined by solid-phase microextraction (SPME), followed by gas chromatography coupled with a mass spectrometer (GC/MS), using a targeted and untargeted approach, have allowed the identification of fifty-nine volatile compounds, among which ethyl acetate, amyl/iso-amyl alcohol, ethyl octanoate, benzaldehyde, ethyl decanoate, and phenylethyl alcohol were predominant. The untargeted approach was able to discriminate wines according to their production area and the year of production. As previously described, during the ageing in the barrels, this wine is subjected to a controlled oxidation induced by the formation of a flor velum on the wine surface [38]. When the sugars and nitrogen compounds are depleted, the flor yeasts shift their fermentative metabolism to oxidative, generating several volatile compounds. This oxidative style gives the wine its distinct character, reminiscent of fortified wines, such as Sherry. It is known for its nutty, dried fruit, and caramelized flavours, with hints of spice and a pronounced tanginess.

Regarding the Malvasia delle Lipari wines, the aroma and oral perception profiles of dry apricot, raisin, caramel, and spicy were associated with several volatile organic compounds compared to the same wines obtained with two different yeasts. Among the 43 volatile components found by Muratore et al. [73], ɣ-butyric lactone, α-terpineol, isoamyl alcohols, 2,3-butanediol, and phenyl ethanol were responsive to these perceptions defined using a trained panel of 36 judges. The same authors assigned a role of primary importance to the yeast strain used to carry out fermentation as a biological control of volatile acidity and aroma. Moreover, among the compounds formed after the refining of Malvasia delle Lipari, furanic derivatives, such as 5-hydroxymethylfurfural and 2-furaldehyde, generated due the hexose and pentose sugar degradation, are involved in the aroma of the Sicilian sweet and fortified wine [41]. In addition, Italian fortified wines can contain a wide range of other VOMs, depending on the specific wine and the winemaking techniques used. Factors, such as the ageing period, type of oak barrel, and storage conditions, can contribute significantly to the volatilomic profile of Italian fortified wines.

## 4. Concluding Remarks

It is well known that the winemaking process improves the generation of several hundred chemical compounds, such as organic acids, terpenoids, pyrazines, higher alcohols, ethyl esters, sulfur compounds, and furanic compounds, which can be considered age, authenticity, and quality markers. Among all the components, the volatile organic compounds are responsible for the complex aromatic patterns and contribute to the sensory perception of the different fortified wines. As explained in the previous chapters, the final quality and typicity of the fortified wine and the related aroma are strictly influenced by the grape quality, the autochthonous microbiota, the fermentation conditions, and the ageing processes. Besides, as discussed before, climate variations can have a significant impact on the production and quality of fortified wines; for instance, climate variations can affect the sugar accumulation in grapes, which is essential for fortified wine production. Warmer climates may lead to higher sugar levels, resulting in wines with higher potential alcohol content. However, excessive heat and drought can also cause dehydration of grapes. Long-term climate changes can challenge traditional practices and require adaptations in vineyard management and winemaking techniques to maintain the quality and style of fortified wines. Nowadays, a more in-depth and complete understanding of vineyard management, the biochemistry of grape-juice fermentation, and the chemistry of wine ageing are essential for assisting the wine business by supporting conventional local winemaker empirical knowledge. A comprehensive understanding must also be addressed for metabolomics and volatolomics in wine analysis to avoid the adulteration of these wines, which are considered niche products with territorial connotations. The large volume of data provided by volatolomics analysis of wine volatile compounds presents a powerful tool to evaluate different aspects in the oenological context for the recognition of the authenticity or geographical origin of the product. Moreover, emerging wine regions around the world have begun producing fortified wines. For example, the Rutherglen region, known as “the fortified wine capital of Australia”, or South Africa, are exploring fortified wine production, experimenting with different grape varieties and styles. The fingerprinting of these wines remains unexplored in the scientific literature.

## Figures and Tables

**Table 1:** The most important aromatic compounds found in Madeira and Porto wines and their respective odour descriptor and threshold (OT) [48,53,56].

VOMs	Structure	Odour Descriptor	OT (µg/L)
α-Terpeniol	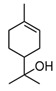	Warm peppery, mildly earthy, musty woody	110
Linalool	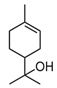	Citrus, floral, fruity, green, muscat, sweet	15
β-Damascenone	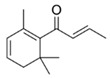	Sweet, exotic flowers, stewed apple	4
β-Ionone	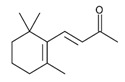	Violet, rose	0.09
Acetaldehyde	CH_3_CHO	Apple	100
2-Nonenal isomer	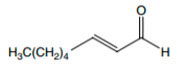	Green, fatty	3
Methional	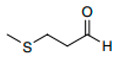	Cooked potato, cabbage	0.5
Phenylacetaldehyde	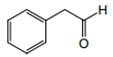	Floral, honey	1
Sotolon	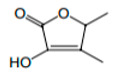	Curry, seasoning	8
γ-Butyrolactone	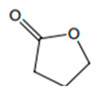	Caramel, sweet	-
*cis*-oak lactone	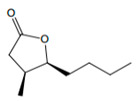	Coconut	25
*trans*-oak lactone	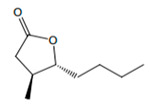	Coconut	110
1,1-Diethoxyethane	CH_3_CH(OCH_2_CH_3_)_2_	Green fruit	1400
Dioxolane and dioxane isomers	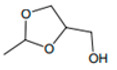	Port-like, sweet	100,000
